# Sequential information processing in persuasion

**DOI:** 10.3389/fpsyg.2022.902230

**Published:** 2022-09-06

**Authors:** Roman Linne, Jannis Hildebrandt, Gerd Bohner, Hans-Peter Erb

**Affiliations:** ^1^Faculty of Humanities and Social Sciences, Helmut Schmidt University, Hamburg, Germany; ^2^Department of Psychology, Bielefeld University, Bielefeld, Germany

**Keywords:** persuasion, social influence, sequence, order effects, assimilation, contrast, social judgment

## Abstract

We present a theory of sequential information processing in persuasion (SIP). It extends assumptions of the heuristic-systematic model, in particular the idea that information encountered early in a persuasion situation may affect the processing of subsequent information. SIP also builds on the abstraction from content-related dichotomies in accord with the parametric unimodel of social judgment. SIP features one constitutional axiom and three main postulates: (A) Persuasion is the sequential processing of information that is relevant to judgment formation. (1) Inferences drawn from initial information may bias the processing of subsequent information if they are either activated rules or valence expectations that are relevant to the subsequent information. (2) Inferences drawn from initial information are resistant to change. Thus, the interpretation of subsequent information is assimilated to inferences drawn from the initial information. Or, if assimilation is impossible, contrast effects occur. (3) The overall effect of a persuasion attempt corresponds to the recipient’s judgment at the moment the processing of information is terminated. We illustrate how our predictions for assimilation and contrast effects may be tested by presenting results from an experiment (*N* = 216) in which we presented exactly the same arguments but varied the processing sequence. We discuss theoretical and applied implications of sequence effects for persuasion phenomena, as well as challenges for further research developing and testing the theory.

## Introduction

Social influence involves all processes resulting in an individual’s attitudes, beliefs, cognitions, and behaviors being changed as a consequence of the intentional or unintentional doings of others (Cialdini and Griskevicius, 2010). One important area of social influence research deals with persuasion, that is, the attempt to change other individuals’ attitudes, usually by presenting arguments in favor of (or against) a given attitude object (e.g., Petty and Cacioppo, 1986; Chen and Chaiken, 1999). During its long history, persuasion research has emphasized the more static aspects of an attempt at persuasion (what are the arguments, the context, the cues), thereby focusing less on the more dynamic aspects (how aspects of the attempt at persuasion interact, depending on their position within a message). In order to place these dynamic aspects at center stage, we present a framework of sequential information processing (SIP) in persuasion.

An experiment by Erb et al. (1998) may serve to illustrate such dynamic effects: Participants received arguments stating that a traffic construction project would benefit their community. According to pilot data these arguments (judged on their own) were moderately strong. In the main experiment, however, some participants had been told that the arguments came from a majority source, others, that they came from a minority source. Participants in the majority condition judged the arguments to be more convincing and were more persuaded overall than participants in the minority condition. Importantly, this source effect was not obtained in other conditions, where information about the source was presented only *after* the arguments.

These findings underline one fact important to persuasion: Sequence matters. The same argument may be perceived as weak, strong, or anything in between, depending on what initial piece of information (POI) has preceded it. Thus, if the recipients learned this additional POI subsequently to the arguments rather than initially, the arguments would necessarily be perceived differently and thus have a different persuasive impact.

Usually, attempts at persuasion entail multiple POIs. We define POIs as all aspects involved in an attempt at persuasion that are subjectively relevant for a recipient’s judgment, including arguments, source information, and context factors. The impact of these POIs is not limited to exerting a separate, static influence toward the final judgment. Rather, they also interact dynamically: Inferences drawn from initial POIs may influence the impact of subsequent POIs within sequential persuasion (e.g., Chen et al., 1996). On the one hand, there may be biased processing of a subsequent POI in line with inferences drawn from an initially received POI (i.e., assimilation; Chen et al., 1996). On the other hand, the evaluation of a subsequent POI may also be contrasted with expectations that are previously held or activated by a previous POI (Bohner et al., 2002). It is important to note that dynamic effects are less a function of the POIs’ (objective) content but rather of the inferences individuals draw from them. A subsequent POI may be interpreted in line with the inferences an individual has drawn from an initial POI. What exactly these inferences are, however, may vary between recipients. Depending on pre-existing attitudes or personality traits, recipients may draw different inferences from the same POI, thus also changing the initial POI’s impact for subsequent POIs. When we speak of an initial POI’s impact in the course of this paper, we always mean the impact of a recipient’s inferences drawn from an initial POI.

In order to describe sequence effects, SIP draws from persuasion theories describing attitude change as the result of either two different processes (Petty and Cacioppo, 1986; Chen and Chaiken, 1999) or a single process (Erb et al., 2003). Specifically, SIP refers to the heuristic-systematic model (HSM, Chaiken et al., 1989) and the parametric unimodel of persuasion (PUM; Kruglanski and Thompson, 1999; for a discussion see Bohner and Siebler, 1999). Although dynamic sequence effects in persuasion may not have been examined to the degree we deem necessary, both HSM and PUM have incorporated specific assumptions regarding the interplay of early and late information within a persuasion setting.

The present article has four primary goals: First, we present a general framework explaining sequential information processing in persuasion (SIP), second, we examine where sequence effects have already been observed in persuasion, attitude, and judgment research, third, we describe and discuss first empirical evidence from an illustrative experiment, and fourth, we discuss possible future directions of research based on SIP.

## Heuristic-systematic model and parametric unimodel of persuasion: Differences and commonalities

Proponents of dual-process models like the HSM describe attitude change as the result of two qualitatively different processes (Chaiken et al., 1989). According to the HSM, these are *heuristic processing* and *systematic processing*. In cases where recipients’ motivation or processing capacity is low, they primarily use heuristic processing. That is, when forming an attitude, they use heuristics, which are exogenous to the message’s content, such as “experts are usually correct” or “the majority’s view is most likely valid.” Therefore, heuristic cues that are easy to process (e.g., the source is described as an expert) are particularly influential. However, when recipients are highly motivated and have sufficient capacity, they will also use systematic processing by engaging with a persuasive message’s content, that is, the presented arguments and their implications.

Authors of the PUM (Kruglanski and Thompson, 1999), by contrast, conceive of attitude change as a single process, abandoning the distinction between heuristic and systematic processing. Instead, continuous parameters are presumed to predict the influence of information, which serves as *evidence* for recipients to reach a conclusion and form an attitude judgment. According to the PUM, it is not relevant whether a given POI constitutes an argument or a heuristic cue. Rather, the impact of a POI depends on (1) its processing difficulty, (2) the recipient’s effort dedicated to the processing of this POI, (3) its perceived relevance for forming a judgment, and (4) its relative position in the sequence of processing. For example, if information about source expertise (i.e., a cue) could only be gained by reading a 10-page CV it would probably not be very influential in situations where motivation or capacity were low (Erb et al., 2003). Similarly, if relevant information were presented late in the persuasion sequence, it would be less influential under low processing effort because recipients may have terminated processing already (Erb et al., 2003).

Despite their distinctive focus on one versus two processes, HSM and PUM also have many assumptions in common. Firstly, both propose a continuum of processing effort. That is, the cognitive effort recipients invest may vary between two extremes: processing only few POIs and processing everything that may be even barely relevant to the final judgment. Secondly, according to both models, the amount of processing depends on recipients’ processing motivation and cognitive capacity (Bohner et al., 1992; Kruglanski and Thompson, 1999). If motivation or capacity is low, recipients will form their judgment based on little, easily accessible information. If, however, recipients’ motivation is high and they also have sufficient capacity, they will process both more—and more complex—information (Petty et al., 1981; Bohner et al., 1992, 1998; Darke et al., 1998; Kruglanski and Thompson, 1999). Thirdly, both models include the notion of specific processing motives, such as striving for accuracy or impression management (Chen et al., 1996).

## Sequence-related aspects of heuristic-systematic model and parametric unimodel of persuasion

The *biased-processing hypothesis* of the HSM holds that factors exogenous to the message content can bias the *subsequent* processing of message arguments. Such factors include the communicator’s credibility (Chaiken and Maheswaran, 1994), recipients’ mood state (Petty et al., 1993; Bohner et al., 1994), consensus among proponents (Darke et al., 1998; Erb et al., 1998), and others (e.g., Ziegler et al., 2005). Biased processing requires that (a) the biasing factor precede the to-be-biased information in the persuasion sequence and (b) the to-be-biased information be sufficiently ambiguous to allow for subjective interpretations based on the first pre-judgment formed in response to the biasing factor. To distinguish a biasing effect from a direct (heuristic) effect, three criteria apply: (1) Cognitive responses, which may be assessed *via* a thought-listing procedure (Greenwald, 1968; Petty and Cacioppo, 1986), reflect the valence of the biasing factor; (2) these cognitive responses determine the final attitude judgment; (3) the effect disappears under the condition that recipients encounter the biasing factor only after the to-be-biased information (Erb et al., 2007). Thus, the sequence in which the POIs are presented is regarded to be crucial for the (non-)occurrence of biasing effects.

The HSM includes hypotheses regarding the interplay of heuristic and systematic information under high processing effort (Bohner et al., 1995). These are relevant to SIP because they specify potential interactions of POIs in a persuasion context. According to the *additivity hypothesis*, cues and arguments may exert influence independent of one another. For example, if a friendly source puts forward strong arguments, the cue (=friendly source) and the arguments (=strong) are both persuasive. Rather than interacting, the persuasive effects of the POIs add up to an overall effect of persuasion. A special case is that the specific impact of a given POI may also be zero (see the HSM’s *attenuation hypothesis*; Chaiken and Maheswaran, 1994). Two further hypotheses emphasize interaction effects, that is, effects of one POI changing the impact of other POIs in a non-linear manner. Besides the notion of assimilative processing described in the *bias hypothesis* (see above; see also Bohner et al., 2002; Tormala and Clarkson, 2007), the HSM includes the notion of interaction between POIs producing contrast effects. According to the HSM’s *contrast hypothesis* (Bohner et al., 1995), heuristic-based expectations may result in a contrasting interpretation of systematically processed information. For example, the heuristic “experts are usually correct” may lead to the expectation that a source which is perceived to be an expert will deliver convincing arguments. If motivation and processing capacity are high, a recipient will compare the argument to this expectation, and if the arguments do not match this standard, will evaluate them more negatively (see Bohner et al., 2002).

Especially the latter two HSM hypotheses are of particular importance for SIP. The dynamic interaction of information described in the bias and contrast hypotheses is based on the notion that the effect of one POI is dependent on a preceding POI, suggesting that an overall effect is not sequence-invariant. For example, if a contrasting interpretation of a given argument requires a cue-based expectation, then without the cue preceding the argument there would be no contrast.

Within the PUM, however, exogenous factors and message content form part of the more general category of persuasive evidence and are thus assumed to be functionally equivalent in the persuasion sequence. Accordingly, any kind of evidence may interact with any other evidence. It has indeed been demonstrated that a message argument, either weak or strong, presented upfront in the persuasion sequence functions in the same way as exogenous factors. The argument may bias the processing of subsequently presented further arguments as well as the processing of exogenous factors such as information on the communicator’s expertise (Erb et al., 2007).

Thus, the PUM includes a notion of sequence effects in persuasion. However, in contrast to the HSM, the PUM does not include specific assumptions regarding the interplay of different types of POI. Nonetheless, the basic premises of the PUM may be integrated with the more specific interaction hypotheses drawn from the HSM, as we outline in the sections below.

## Sequential information processing

Our SIP model is intended to provide a framework for explaining dynamic sequence effects in persuasion. In order to achieve this, we integrate aspects of previous persuasion models into one single model. With respect to the question of one versus two processes, we adopt the assumption of the PUM that there is no theoretically relevant difference between types of POIs (”heuristic cue” vs. “argument”). From the HSM’s co-occurrence hypotheses, we draw inspiration regarding potential interaction effects between POIs.

**FIGURE 1:**
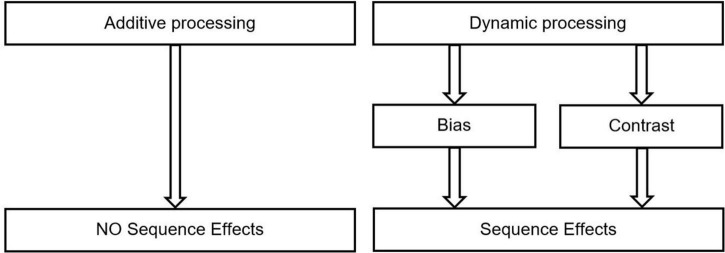
Possible interplays of different pieces of information (POIs) in a persuasion attempt.

The basic assumption behind SIP is that within a process of persuasion, POIs dynamically interact with one another. Thus, POIs are contributing to an overall effect of persuasion not only in an additive way (as in “two arguments are twice as good as one”), but also in an interactive way (POI 1 may boost or discredit the interpretation of POI 2; see Figure 1). Besides their content, the relative position within a persuasive message becomes important. Furthermore, SIP primarily focuses on sequence-related aspects of persuasion. Following Anderson (1981), we assume that “in everyday life, information integration is a sequential process. Information is received a piece at a time and integrated into a continuously evolving impression” (p. 144). Thus, we preface more specific assumptions regarding POI-interaction with the axiom that persuasion is the sequential processing of POIs. Although this insight has guided persuasion research for decades, other areas of research have preferred the simultaneous presentation of information; however, a sequential presentation may not only better reflect humans’ natural judgment processes but may also result in more accurate judgments when objective standards are available (Luan et al., 2020).

We assume that initial POIs may bias the processing of subsequent POIs by increasing the cognitive accessibility of certain inferences regarding the subsequent POI (Erb et al., 1998; Bohner et al., 2002, 2003). Our theoretical generalization refers to two possible mechanisms that may drive sequence effects: (1) As proposed in the PUM, individuals may draw quasi-syllogistic inferences describing the specific thematic interplay of the POIs. An initial POI may provide a premise from which recipients draw a conclusion about the subsequent POI. Thus, POIs processed earlier and conclusions based on this information serve as the basis for later inferences. For example, Bohner et al. (2003) presented participants the (initial) POI that a restaurant served dishes made only from fresh ingredients, followed by the (subsequent) POI that the restaurant’s menu contained only a small selection of dishes (vs. the unrelated information that the restaurant had no outdoor seating). The major premise, the belief that “restaurants featuring a small selection of dishes serve fresh food” linked the POIs, which resulted in a more favorable processing of the otherwise negative information that the restaurant offers only a small selection.

(2) Even syllogistically unrelated POIs may influence each other. In this case, the valence-related content of an initial POI alone may instigate biased processing of subsequent POIs. Content-related aspects (i.e., is the argument a pro or a contra argument? is it rather neutral or extreme?), as well as aspects regarding the recipient (e.g., a good or bad current mood state, see Schwarz et al., 1991) may all serve as potentially influential POIs. The initial POI may change a recipient’s attitude toward the attitude object, resulting in a positive hypothesis-testing of the subsequent POI, or it may change the recipient’s expectation regarding the source’s position (Clark et al., 2013), thus, the expectation regarding subsequent POIs. If, for example, the initial POI refers positively to an attitude object, this creates the expectation that subsequent POIs about this object will also be positive.

The inferences evoked from the initial POI need to be subjectively relevant for the interpretation of the subsequent POI. Otherwise, there is no biased processing of the subsequent POI. In this case, the two POIs do not interact and therefore the processing sequence is irrelevant to the overall judgment of the attitude object. For example, we would assume that the information that a restaurant was lacking dedicated customer parking would not bias the interpretation of the subsequent statement that the same restaurant used fresh ingredients only (Bohner et al., 2003).

Sequential information processing follows the notion that earlier evoked inferences (i.e., as a result of the initial POI) are resistant to change (*inertia assumption*). Therefore, if recipients find it possible, the subsequent POI is assimilated to the inferences drawn from the initial POI (Chaiken and Maheswaran, 1994), but not vice versa, as it is far less likely that inferences drawn from subsequent POIs result in a revision of the interpretation of already processed initial POIs. This is because greater cognitive effort is required to adjust or abandon the inferences drawn first than to interpret subsequent POIs in light of the initial POI (Kruglanski and Thompson, 1999). This assumption is also in line with the notion of recipients using motivated reasoning (e.g., Strickland et al., 2011) to defend early conclusions against new evidence. However, factors exogenous to the message content (e.g., a recipient’s high motivation) may alleviate this effect, resulting in recipients showing a stronger tendency to update their judgments in a Bayesian fashion.

Sometimes it is impossible for recipients to assimilate the interpretation of subsequent POIs to earlier inferences because of obvious contradictions. Then, contrast effects in the interpretation of the subsequent POI occur, resulting in it being interpreted in the direction opposite to the initial POI (Bohner et al., 2002). One possible explanation for the occurrence of assimilation versus contrast is feature overlap between POIs (Tversky, 1977; Herr et al., 1983). Accordingly, a high degree of overlap (e.g., a shared super category, Wänke et al., 2001; value overlap, Chien et al., 2010) results in assimilation, whereas a low degree or lack of overlap results in contrast effects. Thus, we propose that a larger discrepancy between the initial POI and the subsequent POI increases the likelihood of contrast (vs. assimilative) effects. However, making precise threshold predictions about the exact degree of discrepancy between POIs 1 and 2 that is necessary to reliably produce contrast will require further research (for our initial demonstration of contrast effects according to SIP, see the illustrative experiment below).

The overall effect of a persuasion attempt corresponds to a recipient’s judgment at the moment they terminate the processing of POIs. Recipients continuously update their judgment of a given object in light of novel POIs: The judgment they have arrived at when they stop processing new POIs (no matter for what reason) constitutes their final evaluation (see Schwarz and Bohner, 2001). The interpretation of any subsequent POI is a function of the inferences drawn from prior POIs. Thus, a POI’s impact for an overall judgment is also a function of previously received POIs. Consequently, the overall judgment is a function of dynamic POI-interaction.

There are several theoretical accounts describing how POIs can be integrated for an overall judgment. For example, Anderson (1981) described information integration as a sequential process in which a number of POIs are weighted, added, or averaged to reach a judgment. According to his “attention decrement” assumption, recipients pay less attention to successive POIs (see also Erb et al., 2003), thereby giving later POIs less weight in a judgment. Other conceptualizations assume that information integration depends on the mode for encoding and processing evidence (Hogarth and Einhorn, 1992) or on the nature of the task/environmental structures (e.g., Juslin et al., 2008). Given that the focus of this article is on POI interaction rather than a computation of information integration, we limit our prediction to the assumption that the overall judgment corresponds to a recipient’s judgment at the moment the processing of POIs is terminated.

The assumption that sequence effects play an important role in processing persuasive messages is not new. For example, Hogarth and Einhorn (1992) proposed a general anchoring-and-adjustment model (see also Epley and Gilovich, 2006) of belief-updating with the aim to predict sequence effects. Despite being a broad approach to explain information-processing, the model can also be applied to persuasion. Specifically, the authors examined the conditions under which primacy, recency (Murdock, 1962; Anderson, 1981), or no sequence effects would occur in a persuasive message. Although the purpose of the model is similar to the purpose of SIP, their foci differ. Hogarth and Einhorn distinguish between two models of judgment: The subsequent POIs are interpreted in the light of the deviation from the preceding anchor (averaging model) or as positive or negative evidence for the hypothesis (adding model). Consequently, Hogarth and Einhorn focused on predicting order effects that arise from information processing strategies (whether the overall judgment is reported once all POIs have been received or is updated stepwise) interacting with task characteristics (simple vs. complex) and information integration in POI-processing. With SIP, on the other hand, we rather focus on dynamic POI interaction, that is, how sequence relates to the impact of inferences drawn from one POI on a subsequent POI. Furthermore, predictions also vary: Whereas Hogarth and Einhorn predict different modes of adding and averaging information for an overall effect, SIP also predicts contrast effects under specific circumstances.

### Sequential information processing postulates

The current version of the SIP framework can be summarized in one axiom (A), defining its scope, and three postulates (1–3) regarding the dynamics of sequential persuasion.

(A) Persuasion is the sequential processing of information that is relevant to judgment formation.

(1) Inferences drawn from initial POIs may bias the processing of subsequent POIs.
  a. Inferences drawn from the initial POI may either (i) activate logical rules (syllogisms) or (ii) expectations regarding the valence-related content (pro or contra, extremity) of the subsequent POI. If the inferences evoked by the initial POI are relevant to the processing of the subsequent POI, the subsequent POI will exert a different impact on the final judgment than (b) if the initial POI is not relevant. Order effects (judgments vary between reception of POI A → B vs. B → A) occur only if an initial POI is relevant to the processing of a subsequent POI.

(2) Expectations and inferences drawn from information provided in early stages of the persuasion process are comparatively resistant to change.
  a. Thus, the interpretation of subsequent POIs is assimilated to inferences drawn from the initial POI.
  b. If recipients find it impossible to assimilate the interpretation of subsequent POIs, contrast effects occur.

(3) The overall effect of a persuasion attempt corresponds to a recipient’s judgment at the moment the processing is terminated.
  a. Recipients continuously update their judgment in light of novel POIs: the judgment they have arrived at when they stop processing new POIs constitutes their overall evaluation.
  b. The interpretation of any subsequent POI is a function of the inferences drawn from prior POIs. Thus, the overall judgment is a function of dynamic POI-interaction.

## An illustrative experiment of contrast effects as a result of sequential processing

As a first empirical examination of SIP, we present an experiment designed to test valence-based assimilation and contrast effects as laid out in Postulate 2. This is not meant to be seen as an exhaustive test of our postulates, but rather as a first illustration of SIP research and a first test of the notion of contrast as a potential outcome of sequential processing. Above, we hypothesized that the interpretation of subsequent arguments (POIs) would be assimilated to inferences drawn from initial arguments. However, if the valence of subsequent arguments (i.e., pro vs. contra, rather neutral vs. extreme) diverges from that of the initial argument to such a degree that recipients find assimilation impossible, interpretation of the subsequent arguments will be contrasted with the initial argument.

Therefore, we varied the stance of the initial argument: Recipients received either an argument in favor (pro) or against (contra) the attitude object, which was the abolition of cash. Additionally, we also varied whether the three subsequent POIs were either all pro or all contra arguments. Finally, these subsequent arguments were presented in two inverse sequences: from most extreme (pro or contra) to neutral, or vice versa.

As of yet, we are not certain which degree of discrepancy between the initial and the subsequent POI is necessary to produce contrast. Nonetheless we deemed it likely that a larger discrepancy would evoke the perception of information-integration being impossible. Thus, we predicted that an initial contra (pro) argument, directly followed by pro (contra) arguments would lead to a contrast effect. However, this effect should only occur if the most discrepant (vs. neutral) argument was presented immediately after the initial argument. We hypothesized that recipients who received this specific sequence of arguments would report the most positive (negative) attitudes (vs. recipients in all other conditions). Thus, we examined whether contrast effects would be caused solely by the sequence in which otherwise identical information is presented. In all other conditions we predicted additive integration of initial and subsequent arguments into an overall attitude. Table 1 depicts the hypothesized effects for all experimental conditions in the form of an exhaustive contrast pattern.

**TABLE 1:** Design and results.

	Initial argument							
	contra				pro			
Sequence of subsequent arguments	contra ↓ neutral	neutral ↓ contra	pro ↓ neutral	neutral ↓ pro	contra ↓ neutral	neutral ↓ contra	pro ↓ neutral	neutral ↓ pro
1. Argument	contra	contra	contra	contra	pro	pro	pro	pro
2. Argument	contra-	neutral	pro+	neutral	contra-	neutral	pro+	neutral
3. Argument	contra	contra	pro	pro	contra	contra	pro	pro
4. Argument	neutral	contra-	neutral	pro+	neutral	contra-	neutral	pro+
Predicted pattern of attitudes	neg.	neg.	**very pos.**	neutral	**very neg.**	neutral	pos.	pos.
Contrast weights	−1	−1	**+2**	0	**−2**	0	+1	+1
Attitudes	3.67	3.75	**4.43**	3.83	**4.02**	4.14	4.46	4.29
*M* (*SD*)	(1.50)	(1.22)	**(1.75)**	(1.57)	**(1.40)**	(1.77)	(1.13)	(1.27)

*N* = 206; contra- = a strong contra argument; pro+ = a strong pro argument; attitude scale ranges from 1 to 7; a higher value indicates a more positive attitude; contrast effects in bold characters.

## Materials and methods

### Design and hypothesis

Participants were randomly assigned to the conditions of a 2 (initial argument: pro vs. contra) × 2 (subsequent arguments: pro vs. contra) × 2 (sequence of the subsequent arguments: from extreme to neutral vs. from neutral to extreme) design (see Table 1).

### Participants

Our final sample consisted of 206 participants (72 male and 134 female; mostly students).^1^ Participants’ age varied between 18 and 57 years (*M* = 24.32, *SD* = 4.60). With this sample size, the statistical power (1 − *β*) for detecting a medium-sized contrast effect (*f* = 0.25) at α = 0.05 within a 2 × 2 × 2 ANOVA design was greater than 0.94 (Faul et al., 2007).

### Attitude object

The attitude object was introduced as “the (almost) exclusive limitation of all payment transactions to EC and credit cards, online banking, or *via* mobile banking apps.” Because pilot testing (*N* = 78) had shown that attitudes toward the abolition of cash were moderate (*M* = 3.96, *SD* = 2.37, on a scale from 1 = *very negative* to 9 = *very positive*) it seemed likely that both positive and negative attitude change would be observable.

### Persuasive message

The persuasive message contained 4 arguments (e.g., “The risk of cyber-attacks and credit card fraud would increase dramatically, for which the police and other authorities would be unprepared due to a lack of qualified personnel and other resources”). In a pilot test, 40 arguments had been rated for stance (“For each argument, please state the extent to which you think it speaks for or against the abolition of cash;” 1 = *argues against*; 5 = *neutral*; 9 = *argues in favor*) and persuasiveness (“For each argument, please state how persuasive you find it;” 1 = *not at all*; 9 = *very*); arguments for the experimental conditions were selected based on stance (pro, contra, or neutral) and persuasive power (strong, medium, or weak) as required by the design (for details, see Appendix).

### Procedure and dependent variable

The experiment was conducted in a paper-pencil format and in individual laboratory sessions. Participants learned that we were interested in their opinion on the abolition of cash. Next, they received a sequence of arguments. Depending on the initial-argument condition, participants first received either a pro or a contra argument about the abolition of cash. Then, depending on the subsequent-arguments condition, they read three further arguments that were either pro or contra. Depending on the sequence condition, the three subsequent arguments went from most extreme to neutral or vice versa. After each argument participants were asked to write down their current thoughts into a text box (with no restrictions given). Next, participants reported their attitude toward the abolition of cash, which constituted our main dependent variable, on five 7-point semantic-differential items (*bad-good*, *meaningless-meaningful*, *useless-useful*, *undesirable-desirable*, and *impractical-practical*). Participants’ answers were averaged into an attitude index that ranged from 1 = *very negative* to 7 = *very positive* (Cronbach’s α = 0.91).^2^ Participants then reported sociodemographic data (age, gender, German language skills, and area of study or employment status). Finally, participants were thanked, debriefed, and rewarded with a chocolate bar.

## Results and discussion

The overall pattern of participants’ attitudes is shown in Table 1. We conducted an *a-priori* contrast analysis to test our main hypothesis (see Table 1 for contrast weights). Furthermore, we tested whether the observed data showed any residual between-condition effects beyond this contrast (see Abelson and Prentice, 1997). Results supported our hypothesis. The focal contrast analysis was significant, *F*(1,204) = 4.58, *p* = 0.034, *R*^2^ = 0.022, whereas the analysis of residual between-condition effects was not, *F*(6,198) = 0.55, *p* = 0.77, Δ*R*^2^ = 0.016.

It can be seen in Table 1 that, as predicted, the initial contra argument being directly followed by the most discrepant subsequent pro argument resulted in attitudes being the most positive (*M* = 4.43, *SD* = 1.75) of all conditions that included the initial contra argument (*M* = 3.92, *SD* = 1.51). Also as predicted, the initial pro argument being directly followed by the most discrepant contra argument resulted in attitudes being the least positive (*M* = 4.02, *SD* = 1.40) of all conditions with the positive initial argument (*M* = 4.22, *SD* = 1.41). In all other conditions, the predicted assimilation effects were evident. It should be noted that these conditions featured the exact same arguments as the contrast conditions, but in a different sequence. Results strongly indicate that in the case of a high-contrast sequence, recipients find it impossible to assimilate the subsequent POIs (e.g., arguments) to the valence expectation based on the first POI. Instead, the considerable discrepancy between the valences of the initial POI and the immediately subsequent POI leads to an overall attitude more in line with the second POI. However, when recipients do not experience the strong discrepancy, no contrast but rather assimilation effects occur. Thus, results support our assumptions regarding both assimilation (Postulate 2a) and contrast (Postulate 2b).

In an additional analysis, we correlated the valence of the thoughts participants had written down after each argument^3^ with their overall attitude toward the abolition of cash. This showed a highly significant, medium-sized correlation *r(204)* = 0.326, *p* < 0.001. While the result of this exploratory analysis indicates a relation between general thought-valence and overall judgment, it was not designed to test specific SIP postulates.

The primary aim of this experiment was to provide first empirical evidence for the validity of our model and specifically Postulate 2. We were able to empirically show, for the first time, that assimilation and contrast can occur with the same arguments: Attitudes are a function of sequence. Whereas the current experiment featured sequence effects that were based solely on valences of the POIs, future studies should focus on sequence effects that are driven by more specific syllogistic inferences between POIs. Nonetheless, taken together, our results clearly indicate support for SIP.

One practical implication pertains to communicators who try to persuade their audiences using messages including pro and contra arguments. When crafting a persuasive message, communicators may specifically attempt to use a sequence that induces a contrast experience in their recipients in order to achieve a particularly strong persuasion effect. Thus, they may not only want to consider sequence effects suggested by conversational norms (for example, by presenting critical information in a two-sided message last; Igou and Bless, 2007; see also Hogarth and Einhorn, 1992) but also with respect to position and argument strength. According to the present data, a very strong POI (pro/contra) that the communicator wants to emphasize (i.e., making it more persuasive) should be placed directly after a POI of unambiguous but contrary stance (contra/pro). This particular sequence should result in a contrast effect toward the second POI, influencing recipients in the intended direction.

### Limitations

Of course, the reported experiment is no test of the complete SIP model. Instead, it was designed as an initial test of the SIP model’s assumptions regarding assimilation versus contrast effects (as described in Postulate 2), and presented here to illustrate designs that may be used to test SIP in the future. In order to assess on the overall validity of SIP as a theory, further tests of Postulate 2 and all other postulates will be necessary (see Section “Future research” below).

Furthermore, while the number of participants in the illustrative experiment was sufficient for the contrast analysis, more fine-grained analyses such as a real-time assessment of a changing evaluation of the attitude object may require higher numbers of participants. Indeed, this assessment would have been helpful to enhance our understanding of assimilation versus contrast. We asked for participants’ thoughts about the specific POIs they received sequentially. However, this procedure may have resulted in recipients’ thoughts being tailored toward their understanding of the POIs and less of the attitude object. Thus, insights into a changing evaluation as a function of specific POI-sequences were limited.

Another limitation arises from the experimental design. The initial and subsequent POIs were not counterbalanced. As a consequence, the initial POI is never presented as a subsequent POI and vice versa. Thus, we cannot rule out the possibility that the sequence effects found in the experiment are a result of the specific arguments and their interactions.

Finally, we did not specify exact conditions necessary for contrast. Instead, we maximized differences between POIs in order to elicit contrast. While successful, this approach did not provide hints for an approximation of an assimilation-to-contrast threshold (see Section “Future research” below).

## Future directions

The main objective of future research on SIP is to test the validity of our model and its postulates. Given that the experiment described in this article is primarily illustrative in nature, further empirical testing is necessary. In addition, there are theoretical aspects of the model in need of further specification.

## Future research on sequential information processing

The focus of future research on SIP will be on testing Postulates 1 to 3. There is already much research on bias effects in persuasion (e.g., Bohner et al., 2002). Thus, research on Postulate 1 should address the specific inferences drawn from POI-interactions as well as their relevance to subsequent POIs. More theoretical deliberations on the nature of POI relations (e.g., are there “logical” relations) will be explicated in the following section. From an experimental perspective, however, several critical tests of Postulate 1 seem feasible. For example, a simple design to test the assumption that messages are more persuasive if POIs are related could include several POIs of the same polarity but with varying interrelatedness. Similar to findings of Bohner et al. (2003), who had shown that two-sided messages are more persuasive if there are logical relations between the pro and contra arguments, we would expect the same for one-sided messages. Consider, for example, an advertisement for a chair that contains any sequence of the following three pro arguments: The chair is… (1) comfortable, (2) a designer piece, (3) made of high-quality materials. We would expect that a succession of related pro arguments (i.e., designer piece and high-quality materials) would result in more persuasion than a succession of unrelated (or even mismatching) pro arguments (i.e., comfortable and designer furniture). Going further, biased processing could be tested directly compared to additive processing.

Given that the focus of our SIP model is less on POIs’ objective content and more on the subjective inferences drawn from POIs, any test of the model could be extended by including context factors or personality traits likely to result in recipients drawing different inferences from the same POI, which in turn would change their effect on subsequent POIs.

Postulate 2 represents the main divergence of SIP compared to existing research on sequential effects in information processing (e.g., Hogarth and Einhorn, 1992). As such, replicating the effect found in the experiment presented in this article should be given high priority. More generally, future research should pin down more exactly the conditions of contrast versus assimilative processing. Our illustrative experiment suggests that perception of a strong discrepancy between POIs results in contrast effects and, thus, attitudes more in line with the subsequent POI. But this first venture into empirical data collection was not designed to differentiate between subtler variations of POI discrepancy. Given the highly subjective nature of Postulate 3, stating that contrast is a consequence of the individual’s inability to integrate two POIs, it seems difficult to specify conditions under which contrast occurs *a priori*. As a consequence of methodological aspects (assessment methods, etc.) and variance immanent to the main subject of any psychological study (i.e., humans with all their individual differences and susceptibility to context factors), predictions about precise threshold values are generally rare in social psychology. This also holds for contrast in SIP, where, for example, the threshold for contrast vs. assimilation may vary considerably between recipients. Nonetheless, future research on Postulate 2 should be concerned with narrowing down on the conditions necessary for contrast effects. Starting with extremely discrepant POIs, shown to produce contrast (as in our illustrative experiment), future research could gradually reduce discrepancy in an attempt to find an area of discrepancy in which assimilative processing turns into contrastive processing. Assessment of recipients’ thoughts while processing POIs could allow for a measurement of subjective inability to integrate information.

Support for Postulate 3 requires evidence of continuous updating resulting in the overall judgment being the judgment at the time the processing of POIs stopped. In order to test this, the assessment of judgment must be conducted in real time, that is subsequent to the reception of each POI. In the illustrative experiment, we already measured thought valence toward each subsequent POI. Results showed a moderate relation between thought valence and the overall judgment. Furthermore, some participants’ statements reflected the thought processes assumed to occur during specific POI sequences. For example, after the first contrasting POI, a recipient showed surprise, “I’ve never thought about it, but that’s true. I think that’s good!”, whereas another, in the assimilation condition, expressed agreement, “That is absolutely correct.” Nonetheless, evidence for Postulate 3 requires a more fine-grained analysis and an assessment of the judgment of the attitude object across a sequence of POIs (vs. an assessment of thoughts regarding the specific POIs). This procedure could show the evaluation of the attitude object changing as a function of the inferences drawn from POIs relevant to the recipient. The interpretation of POIs, in turn, of course depends on the processing sequence. For example, after the POI that an expert was arguing in favor of the attitude object, the evaluation should be positive. But then, if the expert presents only pathetically poor arguments, the evaluation should turn negative (i.e., even more negative compared to a condition where a layperson delivers the same poor arguments; see Bohner et al., 2002). Depending on whether the processing was stopped after the first or after the second POI, the overall judgment would be either positive or negative. Given that an on-line assessment itself might affect the inferences drawn from the POIs as well as the overall judgment, this operationalization should be conducted parallel to a condition with an assessment of the overall judgment only.

## Specific inferences between pieces of information: Syllogistic relations or valence-based expectations?

Postulate 1 states that expectations regarding subsequent POIs are a function of either a logical relation (i.e., the initial information presents a logical premise for the processing of a subsequent information) or the valence-related content of the initial information. Different relations between POIs may have different implications for sequence effects.

According to the PUM (persuasive) information is evidence if it provides relevant inferences for a judgment (Kruglanski and Thompson, 1999). That is, it must be relevant in a syllogistic manner; the evidence provides the premise for a conclusion. Translated into SIP terminology, the impact of an initial POI for the processing of subsequent POIs would be based on the logical relation between the POIs. In an experiment by Erb et al. (2007), an initial argument in favor of an infrastructure project biased the processing of the subsequent (ambiguous) information which stated that the source had some experience in traffic planning. Depending on the strength of the initial argument (weak vs. strong), the second POI was evidence for the source’s either moderate or high expertise. Here, the recipients’ interpretations of the subsequent POI were biased because the inference from the initial POI was relevant to the subsequent POI. A high-quality statement about traffic infrastructure implies that the source of the statement is knowledgeable in this area of expertise (i.e., traffic planning), whereas the same initial POI would not imply expertise in other, unrelated areas such as medicine or philosophy (and thus, it would not bias POIs regarding attitude objects from these disciplines).

Dynamic effects in persuasion result in changes in the evaluation of single POIs, and this, in turn, changes their contribution to the overall effect of persuasion. If a positive initial POI provides the premise onto which the conclusion may be reached that a subsequent POI is even more positive (negative) than a stand-alone interpretation would have suggested, the overall effect is stronger than the additive effect of the individual POIs. The logical relation also implies directionality. If there is a logical A → B (but not B → A) relation, interaction effects depend on sequence.

This condition, however, would not apply, if interactions among POIs (biasing or contrast effects) were the result of valence-based expectations. Although the initial POI would again be more influential for the processing of the subsequent POIs (vs. vice versa; inertia assumption), dynamic effects should be based solely on the valence of the initial POI (vs. logical relations with the subsequent POI). If the subsequent POI is ambiguous, we should see assimilative bias; if the subsequent POI is of clearly opposite valence, we should see contrast. However, if the initial and the subsequent POI were displaying the same valence, we should observe no sequence effects.

Closer to our assumptions regarding logical relations, there are findings of expectation effects in previous research. Igou and Bless (2003, 2007) have shown that order effects in persuasion can be a function of expectations based on conversational rules (Grice, 1975). Recipients expect the most important argument to be stated at the beginning of a one-sided message, or at the end of a two-sided message. Therefore, a persuasive impact of the order of arguments depends on recipients’ expectations (e.g., in a one-sided message, the first POI *becomes* more influential) unless conversational rules are being discredited (e.g., by stating the order of arguments was determined at random; Igou and Bless, 2007, Experiment 1). These results suggest that conversational rule-based expectations may bias the perceived importance of an argument (and thus also its persuasiveness) depending on its position in the persuasion sequence. In a similar vein, Wänke (2007) has shown that ambiguous information was processed in line with recipients’ expectations regarding the source’s motives. In an advertising context, recipients assumed that the message would argue in favor of a product, and thus interpreted a message including an ambiguous statement (a body lotion containing “Recitin”) more positively (vs. a message without additional, ambiguous information), which resulted in greater attitude change.

According to SIP, we assume that expectations result in biased hypothesis-testing by recipients. If an initial POI results in recipients forming a specific hypothesis (e.g., the final argument of a two-sided message is the most important one, see Igou and Bless, 2007), they will process subsequent POIs in a way that favors their hypothesis (confirmation bias; Nickerson, 1998). Thus, if the valence of an initial POI creates the expectation that a subsequent POI will express the same valence (i.e., an initial pro argument will be followed by a second pro argument), this by itself may result in biased processing of the subsequent argument: A recipient expects a pro argument and, therefore, interprets a subsequent POI accordingly.

Although we differentiate between logical relation-based and valence-based expectations, we do not assume a dual-process model with qualitatively different assumptions based on the specific relation of information. Either way, we expect assimilation of the subsequent POI, or, if recipients find this impossible, contrast effects. Relations based on valence-expectations and relations based on logic may, however, be descriptions for quantitatively varying degrees of the relation between POIs. Future research is necessary to specify our assumptions regarding the dynamic interactions between POIs as a result of their relations. Our first SIP-inspired study (see above) indicated that, even in the absence of logical relations, the processing of subsequent POIs was biased. Nonetheless, interactions might be dependent on the nature of the expectation drawn from initial POIs (Igou and Bless, 2003, 2007). Thus, future research may focus on sequence effects, varying the nature of the POIs (e.g., unambiguous vs. ambiguous), the nature of their relations (e.g., same valence vs. syllogistic relations) as well as the origin of expectations (e.g., conversational rules) in order to expand the theoretical foundations.

## The role of sequential information processing for applied persuasion

The SIP framework may offer helpful indications for applied persuasion. Knowledge about the sequential interplay of several POIs included in an attempt at persuasion and about the effects of specific inferences drawn from initial POIs for the processing of subsequent POIs may allow for the construction of particularly persuasive messages.

We suggest that specific POIs (e.g., cues) may imply specific inferences regarding aspects of a persuasive message. For example, the information that a source is an expert (vs. a layperson) may lead to the expectation that the source will present convincing (vs. weak) evidence, whereas the information that a source is likable (vs. unlikable) may lead to the expectation of a cooperative (vs. aggressive) argumentation style. Also, specific inferences may also satisfy specific processing motives: The expectation that an expert will present strong arguments may satisfy an accuracy motive (“I agree because the expert is most likely correct”), whereas the expectation that a likable communicator will argue cooperatively may satisfy a connectedness motive (“I agree because it is socially rewarding”; for related evidence, see Linne et al., 2022).

Therefore, not every weak subsequent POI that follows a positive initial POI will result in a violation of expectations. For example, an expert presenting weak arguments would result in a contrast effect because of a violation of the expectancy that arguments would be strong (Bohner et al., 2002). However, in case of a likable communicator presenting weak arguments we would rather predict additive integration of the two elements of the message, that is, instead of contrast there would result a more positive attitude compared to a condition in which an unlikable communicator presents the same weak arguments.

To sum up, we have suggested a framework to examine dynamic sequence effects in persuasion. Furthermore, we were able to present first evidence in favor of our framework. However, there is much to consider in future theorizing and empirical research.

## Data availability statement

The primary data and coding scheme of the reported experiment are available as Supplementary material. Any further inquiries may be directed to the corresponding author.

## Ethics statement

The studies involving human participants were reviewed and approved by Ethics Committee of Bielefeld University (EUB). Written informed consent from the participants’ legal guardian/next of kin was not required to participate in this study in accordance with the national legislation and the institutional requirements.

## Author contributions

JH conducted the original research, designed the experiment, and collected the data (with support from GB). JH and GB performed the statistical analyses. JH and RL wrote first draft of the manuscript. All authors contributed to the formulating the presented theory and manuscript revision and approved the submitted version.

## Footnotes

1 From an initial sample of 216 participants, data from 10 non-native speakers were excluded. Including these participants would not have changed results, with the focal contrast analysis (see Section “Results and discussion”) remaining significant and the analysis of residual between-condition effects remaining non-significant.

2 We also assessed participants’ need for cognition (Bless et al., 1994). However, analyses did not show any relevant effects.

3 Independently rated by two research assistants with high interrater reliabilities, all *r* > 0.84, all *p* < 0.001. The thought-valences of all non-neutral arguments were averaged for analysis.

## Appendix

### Pilot data of the message manipulation

**TABLE A1:** Message manipulation and pilot data.

			Stance	Pers. power
		Argument	*M* (*SD*)	*M* (*SD*)
Initial argument	−	According to current surveys, 80% of Germans are in favor of preserving cash.	3.68 (2.42)	4.85 (2.19)
	+	Only 19% of all payment transactions in Sweden are still made in cash.	6.30 (1.74)	4.10 (2.22)
Subs. argument	−−	The risk of cyber-attacks and credit card fraud would increase dramatically, for which the police and other authorities would be unprepared due to a lack of qualified personnel and other resources.	2.42 (2.25)	6.97 (2.09)
	−	Alternative currencies (precious metals such as gold) would be used for the black market, which could make some products, such as jewelry, more expensive due to increased demand.	3.47 (1.88)	4.93 (1.97)
	++	Crimes could be better monitored by the state. This would make conditions for the black market (drug dealers, thieves, etc.), corruption, money laundering, and robberies much more difficult.	7.73 (1.38)	6.20 (2.15)
	+	Computer scientists would become even more in demand due to the increasing demand for developing and improving online payment systems.	6.50 (1.77)	4.05 (2.24)
	o	The first coins were issued as a payment method in the empire of the Lydians between 650 and 600 BC. Paper money was invented in the 11th century in China during the Song Dynasty.	4.87 (1.56)	2.79 (1.91)

N = 78; −− = used as a strong contra argument, − = used as a contra argument, o = used as a neutral argument, + = used as a pro argument, ++ = used as a strong pro argument; Stance = rating of pro-contra: scale reaches from 1 = argues against (the abolition of cash) to 9 = argues in favor (for the abolition of cash); Pers. power = rating of persuasive power: scale reaches from 1 = not at all to 9 = very.
