# Supplementary material for: Sequential information processing in persuasion
Source: Front Psychol. 2022 Sep 6;13:902230. doi: 10.3389/fpsyg.2022.902230 (PMC9487525; doi:10.3389/fpsyg.2022.902230)
Supplement: Supplementary file 1 [file Data_Sheet_1.docx]

**Supplementary Material**

**Primary Data**

***Primary Data of the Main Study (ASCII Data File Format)***

001343426153243462232671211000110

002814414141114144114541321000110

003534535332235224224251271000120

004513314222222222222252251000140

005667457111111121111111241000150

006211112111111331117661261000120

007357556133222212223661261000120

008324423522322423324332211000160

009411334253123312114261291000120

010723312231111111112271341000120

011456635111111111111172331000110

012200006000112221112222211000130

013645646251253523121352212306130

014423525121211211122262251000140

015867767122121213212262301000170

016813415552212215545242251000170

017524451221215213434542181000001

018134431222121221222242191000001

019122214211143521115221241000140

020744446223122263114542282104170

021734433133211331123221231000130

022656636353212521115462241000130

023256364223112211232252312205170

024155446122222433234551191000140

025356754242344646334241181000140

026677777621161274311432311000002

027543546231146545464241242206008

028677777131111131113461201000150

029535435253221323226342231000160

030313324434321233223322271000008

031566666225223336223452301000170

032432224222121311114342271000120

033545545132222433223432241000120

034124426252233334223352241000160

035724423242222332223421281000008

036245646122222321213242212408130

037645525112351151111132222515100

038745546122111232113672221000160

039545456223213321124342271000160

040224333222112213234242222200140

041635537123121432125322261000170

042347567121112223211252191000008

043243445432342355526322261000120

044412212111111111212432251000008

045144546121111111114262261000120

046122213121222322122442261000190

047555336242225125313252231000140

048535422002230323023241201000130

049335537232233342115461201000130

050766657111112172223372212000140

051333336546434223436231262124003

052233337556413113113132342133003

053311111113711111111171352620160

054224647252212111117231261000004

055466777474121214322321262624110

056564551111111111112352222710120

057411111422322222122261251000160

058155736342523236555261211000110

059445445255223324231542271000140

060832311133232332434361222605120

061366657142232234431232281000120

062867565221222313333422241000160

063113313244233423333442241000130

064855466243212314124322211000140

065244526442543333213242212200130

066465667111121213337372212300130

067735537111111111111172201000150

068766557154334333334352241000150

069655555132223224126511261000170

070844444711111122112121232903008

071423533173134512114252241000170

072577777233221212225342261000170

073355446152213224113232291000140

074234525154333546532532241000140

075713414233222543112462221000001

076434535343112334334251251000008

077314444111141314214111272205130

078423322365533364336632261000140

079566756232162363224211261000140

080166757112212222111172252100170

081545456123211112124362262104004

082867554111144312112232191000130

083224425522135413123431231000150

084623434245424145114441241000004

085635445352214322224262201000120

086714515312332434334551251000130

087455555345343556654531392916160

088312324242223436446441291000170

089711111111111211111171201000150

090245435234212443226542221000120

091813324123111211112252201000120

092723344232234532213252201000008

093367567112212212121231251000100

094765647132113143247351301000150

095713326343442231113332232103130

096467467122232222113332241000140

097566546546645765756361292827150

098334455466334423443351231000150

099445545521223213133251271000150

100144426222112124337362301000008

101735526343542535432621301000140

102526426122122621113461221000110

103624445222211123112362211000008

104135524123414112224471281000130

105362256642132222111531292100150

106333456402211224115622252300140

107245545421111213121231211000130

108143637222221322133552241000170

109134626252334332224541221000170

110267667432622323442322231000130

111722332253223331223342281000170

112213325222522323231532322004170

113811112433454655445632211000150

114225513243233544334241211000150

115422313223324325424332241000140

116134435241111113122132272602130

117811313133213325442242272600130

118845657122111423124332221000130

119145466111111121112262231000130

120877777111111111111171282605130

121766667122222313223262251000140

122311113111112211111172201000170

123744422222213411211332261000140

124756667242221213224342231000120

125555747122122111114251241000110

126377777131111112112272231000130

127422325722122432222512221000008

128714313435775671234551242322150

129333323243322222223332231000140

130423722275462723677761201000150

131634435122222221112262222105008

132811111143134356335441271000130

133377777631113242213272211000110

134555657132222621114332211000130

135640141121112212112261201000150

136866657121122213122261191000008

137612437152112522123172211000190

138733326345126455333442221000140

139613213244221541124541311000000

140111111735653622222242201000140

141545577243423324536632192100170

142535534222111224233332242900130

143211134652213223114252231000140

144223524743333334136541211000140

145724422555422423224352231000120

146412321243322644324641261000120

147235426222312311216541201000000

148455546233224354123552231000140

149513314111111111111171261000140

150445647321212313242462221000140

151523545233213324322242221000170

152653525532123723324152201000120

153166666243222232125342291000140

154433647225235414333242231000170

155122115366436646343121202003140

156866567622212322313561221000110

157866657223111213223111231000130

158855634121234321113241221000110

159856657254222221122532291000150

160754453533455665226622242608130

161223216232211211113242231000140

162235547211111111112212211000130

163356464243222223442242201000140

164234423211121211213361251000130

165367777121211213212211191000008

166334426135222233124442262304008

167524346262222727234342261000120

168444711474455555755732511000000

169111111465423345322331282103008

170477676232232221113452221000160

171656655132232232323552191000120

172636647264223324344332211000170

173654445332323435323432221000008

174754554122231364136512251000130

175534666732421225115522211000170

176112231262113242114231221000120

177613723222123315223352251000190

178313331111121211122362201000180

179724236232343412433431211000150

180625517111111321113351201000150

181853257242111223324252251000170

182346546232133324233362281000005

183124444622222224333242571000006

184433444265323276236532221000170

185232122242222621223262181000001

186456453232455343345241231000140

187567547116111124334442232620110

188634423342111313223322251000140

189534536222222211121252221000140

190824445323321334345441251000110

191776547142211111111172241000140

192815344122212273121361271000130

193144445244222323323442281000140

194223322242223262123351231000140

195567667554414346555541202215150

196211112111412111111172332230120

197821617111111214223272301000190

198645544111111311111161231000180

199123416111111112211172221000150

200455546222222321113342231000160

201111111130311222114121302626150

202814414145111121114142251000170

203755657145222324135442241000130

204234545332221423231352231000120

205123422221331224432342221000160

206635533364443545343422211000170

207134626133143524113341201000140

208377777232323435343522231000120

209656557223345564337322211000140

210844445252233432335432231000120

211822311145134341114631331000007

212624423363355532226432231000140

213224545253223234346352241000140

214622424334212114242252251000140

215157767111111111112172271000120

216866657123211112112212262105130

***Data Definitions of the Primary Data***

| **Column** | **Variable** | **Coding** |
| --- | --- | --- |
| 1 - 3 | subject ID | 001 - 216 |
| 4 | condition | **initial subsequent sequence**  **argument argument**  1 = negative negative extreme to neutral  2 = negative negative neutral to extreme  3 = negative positive extreme to neutral  4 = negative positive neutral to extreme  5 = positive positive extreme to neutral  6 = positive positive neutral to extreme  7 = positive negative extreme to neutral  8 = positive negative neutral to extreme |
| 5 | DV: bad-good | 1 – 7  0 = missing |
| 6 | DV: meaningless-meaningful |  |
| 7 | DV: useless-useful |  |
| 8 | DV: undesirable-desirable |  |
| 9 | DV: impractical-practical |  |
| 10 | NFC1tbr | 1 – 7  0 = missing  tbr (to be recoded) = reverse coded  [Bless, H., Wänke, M., Bohner, G., & Fellhauer, R. F. (1994). Need for Cognition: Eine Skala zur Erfassung von Engagement und Freude bei Denkaufgaben. *Zeitschrift für Sozialpsychologie*.] |
| 11 | NFC2tbr |  |
| 12 | NFC3tbr |  |
| 13 | NFC4tbr |  |
| 14 | NFC5tbr |  |
| 15 | NFC6tbr |  |
| 16 | NFC7tbr |  |
| 17 | NFC8tbr |  |
| 18 | NFC9tbr |  |
| 19 | NFC10tbr |  |
| 20 | NFC11tbr |  |
| 21 | NFC12tbr |  |
| 22 | NFC13tbr |  |
| 23 | NFC14 |  |
| 24 | sex | 1 = male 2 = female 3 = others |
| 25 – 26 | age | in years |
| 27 | native language | 1 = German 2 = others |
| 28 | foreign language  (if native language = 2) | 1 = Turkish 2 = Russian 3 = Kurdish  4 = Polish 5 = French 6 = Persian, Turkic languages, Arabic, Tamil, Armenian  7 = Croatian 8 = English 0 = missing |
| 29 – 30 | age: beginning to learn German | in years |
| 31 | student at University Bielefeld | 1 = yes 0 = no |
| 32 | area of study | 1 = economics 2 = psychology, sports  3 = law 4 = teaching  5 = STEM 6 = languages  7 = social sciences 8 = sociology, politics  9 = others 0 = missing |
| 33 | other employment | 1 = school 2 = retail 3 = lang. course  4 = healthcare 5 = teaching 6 = law  7 = manager 8 = other university  0 = missing |
